# Association between maternal and child health care and neonatal death in Angola: a secondary analysis of Angola Demographic Health Survey 2015–16

**DOI:** 10.1186/s41182-024-00658-5

**Published:** 2024-11-26

**Authors:** Akiko Saito, Masahide Kondo

**Affiliations:** Department of Health Care Policy and Health Economics, Institute of Medicine, University of Tsukuba, 1-1-1 Tennodai, Tsukuba, Ibaraki 3058577 Japan

**Keywords:** Neonatal mortality, Maternal and child health care, Antenatal care, Postnatal care, Low- and middle-income country, Angola

## Abstract

**Background:**

Neonatal mortality has decreased in Angola; however, it remains high. Quality maternal and child health (MCH) care is key to improving neonatal survival. In Angola, however, the association between neonatal mortality and MCH care has not yet been investigated. Therefore, this study aimed to identify the association between neonatal mortality and MCH services in Angola.

**Methods:**

We used the Angola Demographic Health Survey 2015–16, which is the latest nationally representative dataset of Angola. The associations between early/late neonatal death and MCH care utilization were identified by a multivariable logistic regression analysis, adjusted by the sex of the child, type of residence, wealth index, caesarian section, size of the child at birth and delivery assisted by skilled birth attendant. The individual sample weight, sample strata for sampling errors/design, and cluster number were incorporated in a descriptive and logistic regression analysis to account for the unequal probability sampling in different strata.

**Results:**

The early and late neonatal mortality rates were 22 and 2 per 1000 live births, respectively. We found that having none, one, two or three tetanus injections before the last pregnancy compared to five or more injections, and no postnatal health check for children before discharge were associated with the occurrence of late neonatal death. During the first 2 days after childbirth, no observation of breastfeeding, no counseling on breastfeeding, no counseling on newborn danger signs, no measurement of child body temperature, and no examination of the cord as well as not checking child health before discharge were associated with early neonatal death.

**Conclusions:**

Doses of maternal tetanus vaccination and postnatal child health check before discharge were modifiable factors associated to late neonatal death. Further studies to improve MCH care coverage are needed.

## Background

The United Nation’s Sustainable Development Goals target every country to achieve less than 12 neonatal deaths per 1000 births annually by 2030 [1]. In Angola, neonatal mortality has decreased from 37 estimated in 2000 to 27 estimated in 2021 [2]. However, considerable effort to achieve this target in Angola is still required. Availability and quality of care would be important factors to improve neonatal survival.

World Health Organization (WHO) guidelines recommend evident essential care for pregnant women and newborns to improve birth outcomes and neonatal survival [3–5]. Maternal infection and pregnancy complications may result in severe maternal and child outcomes. The WHO guideline of antenatal care recommends iron supplementation to avoid anemia and any subsequential disorders, and preventive malaria and intestinal parasite drugs in prevalent areas [3]. Blood pressure, urine, and blood samples are taken during pregnancy to detect the risk and occurrence of pregnancy complications, such as hypertensive disorders in pregnancy and diabetes. Tetanus vaccination is also recommended to protect mothers and newborns [6]. WHO guidelines of neonatal care recommend essential neonatal care, which includes appropriate clean and dry cord care, keeping newborns warm, exclusive breastfeeding, and assessment of the newborn for danger signs during postnatal care [4, 5].

Even evident care is known, its effectiveness is not always guaranteed. Insufficient availability and quality of the care reduce its impact and effectiveness [7]. In addition, care is not always available for mothers and children even if they visit a health facility in Angola [8, 9]. Therefore, expected positive correlation between evident maternal and child health (MCH) care and neonatal survival may also be undermined for such reasons.

Previous studies have investigated in association between MCH care and neonatal mortality. Studies conducted in African countries have indicated a correlation between antenatal care (ANC) attendance, facility delivery, and neonatal mortality [10, 11]. Clean postnatal care [11], early initiation of breastfeeding [10, 11] and receiving two or more doses of tetanus toxoid injections [10] are preventive factors of death. However, association between MCH care and neonatal death has not been investigated well in Angola. Therefore, this study aimed to identify the association between MCH care and neonatal mortality in Angola.

## Methods

### Data source

The data were derived from the latest Angola Demographic Health Survey (DHS) 2015–16 dataset, which is publicly available [12]. The Angola DHS 2015–16 is a nationally representative household survey. Women aged 15–49 years old, their children aged under 5 years, and their husband were eligible for the survey [12].

### Sampling and participants

The Angola DHS 2015–16 used three stratified stage sampling strategies. The detailed sampling methodology is described in a previous report [12]. A total of 13,356 mother and child pairs were included in the survey. Among these, mothers of youngest child who had given birth within the 5 years preceding the survey were eligible to answer the ANC and PNC questionnaires. In addition to above criteria, a question regarding tetanus immunization before the last pregnancy was limited to mothers who have received none or one dose of tetanus injection during the last pregnancy and a question regarding child health check before discharge was only for women who have delivered at health facility.

### Dependent variables

We used two binary child clinical variables of early neonatal death (yes = 1, no = 0) and late neonatal death (yes = 1, no = 0). Early neonatal death was defined as death during the day of birth to the 7th day of birth. Late neonatal death was defined as death during the 8th day of birth to the 28th day of birth.

### Independent variables

The following variables of MCH care were examined: number of antenatal care visits (never, 1–3 times, or 4 times or more), number of tetanus injections before the last pregnancy (never, once, twice, three times, four times, five times or more), iron syrup/tablets taken during pregnancy (yes or no), drug for malaria prevention [sulfadoxine and pyrimethamine (SP)/Fansidar] taken during pregnancy (yes or no), drug for intestinal parasites taken during pregnancy (yes or no), blood pressure taken during pregnancy (yes or no), urine sample taken during pregnancy (yes or no), blood sample taken during pregnancy (yes or no), child health checked before discharge (yes or no), breastfeeding observed during the first 2 days after birth (yes or no), counseled about breastfeeding during the first 2 days after birth (yes or no), counseled about newborn danger signs during the first 2 days after birth (yes or no), temperature measurement of the newborn during the first 2 days after birth (yes or no), and examination of the cord during the first 2 days after birth (yes or no). Responses of ‘don’t know’ were classified as ‘never” or “no”.

### Statistical analysis

The association between early and late neonatal death and MCH care provided was identified by a multivariable logistic regression analysis. All analyses were conducted using IBM SPSS 29.0 (IBM Corp., Armonk, NY, USA). SPSS version 29 complex samples package was used to account for the unequal probability sampling in different strata. The individual sample weight, sample strata for sampling errors/design, and cluster number were incorporated in a descriptive and logistic regression analysis [13].

A univariable logistic regression analysis was performed to determine the association between early/late neonatal death and each MCH care. Multivariable logistic regression models with each MCH care variable were estimated for each outcome. Each model was adjusted by six of the following previously reported covariates: child’s sex (girl or boy), place of residence (rural or urban), the wealth index (poorest, poor, middle, richer, or richest) [14, 15], caesarian section (yes or no), delivery assisted by skilled birth attendant (yes or no) and size of child at birth (very large, larger than average, average, smaller than average, very small) [16]. Missing values were excluded from the analysis. Odds ratios (OR) and adjusted odds ratios (aOR), along with the 95% confidence interval (CI), were calculated. Multicollinearity was evaluated by calculating the Variance Inflation Factor for the independent variables, with no values exceeding 5.

## Results

A summary of the studied variables is shown in Table 1. The early neonatal mortality rate was 22 per 1000 births and the late neonatal mortality rate was 2 per 1000 births. The percentage of mothers who never had a tetanus injection during the last pregnancy was 23.4%, that of those who had a tetanus injection once was 19.2%, and that of those who had a tetanus injection twice and more was 56.0%. Among mothers who had none or only one tetanus injection during the last pregnancy, 1.2% had received five times or more tetanus injections before the last pregnancy, and 68.8% had never received a tetanus injection. The percentage of children who had a health check before discharge was 59.6%. During the 2 days after birth, observation of breastfeeding was performed in 32.4% of mothers, counseling on breastfeeding in 39.8% and counseling on newborn danger signs in 33.9%. A total of 34.2% and 37.0% of newborns had their temperature measured and cord examined during the first 2 days after birth, respectively.

**Table 1:** Summary of variables

	*n*	%
Early neonatal death (per 1000 births)		
Yes	293	22.0
No	13,063	978.0
Missing	0	0.0
Late neonatal death (per 1000 births)		
Yes	27	2.0
No	13,330	998.0
Missing	0	0.0
Neonatal death (per 1000 births)		
Yes	321	24.0
No	13,035	976.0
Missing	0	0.0
Sex of child		
Male	6653	49.8
Female	6703	50.2
Missing	0	0.0
Wealth index		
Poorest	2947	22.1
Poorer	3179	23.8
Middle	2963	22.2
Richer	2391	17.9
Richest	1876	14.0
Missing	0	0.0
Type of place of residence		
Urban	8063	60.4
Rural	5293	39.6
Missing	0	0.0
Number of antenatal care visit		
Never	1535	21.5
1–3 times	1655	23.2
4 times or more	3868	54.2
Don’t know	85	1.2
Missing	0	0.0
Number of tetanus injection before the last pregnancy		
Never	2573	68.8
Once	569	15.2
Twice	258	6.9
Three times	123	3.3
Four times	20	0.5
Five times or more	45	1.2
Don’t know	150	4.0
Missing	0	0.0
Number of tetanus injection during the last pregnancy		
Never	1992	23.4
Once	1632	19.2
Twice or more	4756	56.0
Don’t know	115	1.4
Missing	0	0.0
SP/Fansider taken during pregnancy		
Yes	4838	56.9
No	3481	43.1
Don’t know	175	0.0
Missing		
Iron syrup/tablet given during pregnancy		
Yes	6341	74.6
No	2094	24.6
Don’t know	60	0.7
Missing	0	0.0
Drug for intestine parasites given during pregnancy		
Yes	4178	49.2
No	4123	48.5
Don’t know	193	2.3
Missing	0	0.0
Blood pressure taken during pregnancy		
Yes	5998	86.2
No	962	13.8
Missing	0	0.0
Urine sample taken during pregnancy		
Yes	5726	82.3
No	1234	17.7
Missing	0	0.0
Blood sample taken during pregnancy		
Yes	5934	85.3
No	1026	14.7
Missing	0	0.0
Child health checked before discharge		
Yes	2540	59.6
No	1496	35.1
Don’t know	207	4.9
Missing	16	0.4
Observed breastfeeding during 2 days after childbirth		
Yes	2751	32.4
No	5714	67.3
Don’t know	30	0.4
Missing	0	0.0
Counseled on breastfeeding during 2 days after childbirth		
Yes	3377	39.8
No	5079	59.8
Don’t know	39	0.5
Missing	0	0.0
Counseled newborn danger signs during 2 days after childbirth		
Yes	2883	33.9
No	5525	65.0
Don’t know	87	1.0
Missing	0	0.0
Measured temperature of infant during 2 days after childbirth		
Yes	2906	34.2
No	5360	63.1
Don’t know	229	2.7
Missing	0	0.0
Examine cord during 2 days after childbirth		
Yes	3143	37.0
No	5173	60.9
Don’t know	179	2.1
Missing	0	0.0
Caesarian section		
Yes	495	3.7
No	12,833	96.3
Missing	0	0.0
Delivery assisted by skilled birth attendant		
Yes	6511	48.7
No	6846	51.3
Missing	0	0.0
Size of child at birth		
Very large	2322	17.4
Larger than average	1369	10.2
Average	7719	57.8
Smaller than average	572	4.3
Very small	719	5.4
Don’t know	656	4.9
Missing	0	0.0

The associations between MCH care and early neonatal mortality are shown in Table 2. The multivariable logistic regression analysis showed that not checking child health before discharge (aOR: 2.73, 95% CI 1.59–4.68) had a greater odds of early neonatal mortality than child health checked before discharge. No observation of breastfeeding during the 2 days after childbirth (aOR: 2.80, 95% CI 1.47–5.35), no counseling on breastfeeding during the first 2 days after childbirth (aOR: 4.40, 95% CI 2.48–7.82) and no counseling on newborn danger signs during the first 2 days after childbirth (aOR: 3.82, 95% CI 2.08–7.01) were associated with early neonatal mortality. Furthermore, not measuring child body temperature during the first 2 days after childbirth (aOR: 2.08, 95% CI 1.18–3.66) and not examining the cord during the first 2 days after childbirth (aOR: 2.70, 95% CI 1.59–4.58) were associated with early neonatal mortality.

**Table 2:** Association between maternal and child health service and early neonatal mortality

	*n*	%	Odds	95% CI		aOdds	95% CI	
Number of antenatal care visit								
Never	43	2.7	1.96	1.17	3.29	1.71	0.92	3.19
1–3 times	36	2.2	1.61	0.95	2.71	1.46	0.85	2.51
4 times or more	72	1.4	Ref.			Ref.		
Number of tetanus injection before the last pregnancy								
Never	45	2.5	1.39	0.18	10.70	1.05	0.13	8.43
Once	21	2.0	1.09	0.14	8.46	0.83	0.11	6.57
Twice	13	2.4	1.30	0.14	12.02	0.95	0.10	8.90
Three times	6	3.0	1.66	0.15	18.05	1.21	0.12	12.07
Four times	2	2.6	1.44	0.12	16.87	1.31	0.11	15.78
Five times or more	1	1.8	Ref.			Ref.		
SP/Fansider taken during pregnancy								
Yes	72	1.5	Ref.			Ref.		
No	80	2.2	1.48	0.95	2.30	1.28	0.83	1.98
Drug for intestine parasites given during pregnancy								
Yes	105	1.7	Ref.			Ref.		
No	46	2.1	1.53	0.98	2.39	1.28	0.82	1.99
Iron syrup/tablet given during pregnancy								
Yes	59	1.4	Ref.			Ref.		
No	92	2.1	1.31	0.82	2.08	1.06	0.66	1.69
Blood pressure taken during pregnancy								
Yes	88	1.5	Ref.			Ref.		
No	23	2.4	1.62	0.92	2.88	1.39	0.79	2.44
Urine sample taken during pregnancy								
Yes	87	1.5	Ref.			Ref.		
No	24	1.9	1.27	0.74	2.18	1.04	0.59	1.82
Blood sample taken during pregnancy								
Yes	93	1.6	Ref.			Ref.		
No	18	1.7	1.10	0.60	2.01	0.89	0.49	1.63
Child health checked before discharge								
Yes	21	0.8	Ref.			Ref.		
No	53	3.1	3.75	2.05	6.86	2.73	1.59	4.68
Observed breastfeeding during 2 days after childbirth								
Yes	21	0.8	Ref.			Ref.		
No	130	2.3	3.04	1.58	5.84	2.80	1.47	5.35
Counseled on breastfeeding during 2 days after childbirth								
Yes	18	0.5	Ref.			Ref.		
No	133	2.6	4.94	2.61	9.36	4.40	2.48	7.82
Counseled newborn danger signs during 2 days after childbirth								
Yes	15	0.5	Ref.			Ref.		
No	136	2.4	4.61	2.31	9.21	3.82	2.08	7.01
Measured temperature of infant during 2 days after childbirth								
Yes	29	1.0	Ref.			Ref.		
No	122	2.2	2.25	1.30	3.88	2.08	1.18	3.66
Examine cord during 2 days after childbirth								
Yes	126	2.4	Ref.			Ref.		
No	25	0.8	3.06	1.78	5.27	2.70	1.59	4.58

*n* number of early neonatal deaths, *OR* odds ratio, *aOR* adjusted odds ratio, *95% CI* 95% confidential interval

The associations between MCH care and late neonatal mortality are shown in Table 3. The multivariable logistic regression analysis showed that among mothers who received none or only one tetanus injection during the last pregnancy, no tetanus injection before the last pregnancy (aOR: 5.08, 95% CI 4.84–14.00), one injection (aOR: 3.29, 95% CI 1.03–10.55), two injections (aOR: 9.58, 95% CI 1.78–51.58), and three injections (aOR: 14.38, 95% CI 3.68–56.30) was associated with greater odds of late neonatal mortality compared to five or more tetanus injections before the last pregnancy. Not checking child health before discharge (aOR: 6.12, 95% CI 3.12–11.99) was associated with late neonatal mortality.

**Table 3:** Association between maternal and child health service and late neonatal mortality

	*n*	%	Odds	95% CI		aOdds	95% CI	
Number of antenatal care visit								
Never	4	0.2	1.72	0.48	6.15	1.15	0.36	3.65
1–3 times	2	0.1	0.97	0.19	4.84	0.76	0.17	3.38
4 times or more	7	0.1						
Number of tetanus injection before the last pregnancy								
Never	4	0.2	8.74	3.53	21.63	5.08	1.84	14.00
Once	1	0.1	3.68	0.80	16.86	3.29	1.03	10.55
Twice	1	0.3	9.65	1.32	70.75	9.58	1.78	51.58
3 times	1	0.4	15.12	3.37	67.84	14.38	3.68	56.30
4 times	0	0.0	1.00	0.67	1.51	1.41	0.77	2.56
5 times or more	0	0.0						
SP/Fansider taken during pregnancy								
Yes	8	0.2						
No	6	0.2	0.97	0.31	3.09	0.67	0.27	1.65
Drug for intestine parasites given during pregnancy								
Yes	7	0.2						
No	7	0.2	0.92	0.28	2.98	0.58	0.25	1.36
Iron syrup/tablet given during pregnancy								
Yes	10	0.2						
No	3	0.2	1.01	0.31	3.31	0.68	0.26	1.81
Blood pressure taken during pregnancy								
Yes	8	0.1						
No	2	0.2	1.51	0.38	6.04	1.37	0.44	4.26
Urine sample taken during pregnancy								
Yes	9	0.2						
No	1	0.1	0.76	0.15	3.90	0.50	0.14	1.88
Blood sample taken during pregnancy								
Yes	6	0.1						
No	5	0.4	6.01	1.75	20.59	4.22	0.49	36.66
Child health checked before discharge								
Yes	0	0.0						
No	5	0.3	21.21	9.78	46.01	6.12	3.12	11.99
Observed breastfeeding during 2 days after childbirth								
Yes	12	0.2						
No	2	0.1	3.29	0.75	14.38	2.44	0.63	9.48
Counseled on breastfeeding during 2 days after childbirth								
Yes	5	0.1						
No	9	0.2	1.17	0.35	3.87	0.80	0.21	3.12
Counseled newborn danger signs during 2 days after childbirth								
Yes	4	0.1						
No	10	0.2	1.41	0.40	4.98	0.98	0.26	3.76
Measured temperature of infant during 2 days after childbirth								
Yes	2	0.1						
No	12	0.2	3.53	0.82	15.13	2.58	0.62	10.79
Examine cord during 2 days after childbirth								
Yes	3	0.1						
No	10	0.2	1.94	0.55	6.86	1.46	0.36	6.03

*n* number of late neonatal deaths, *OR* odds ratio, *aOR* adjusted odds ratio, *95% CI* 95% confidential interval

## Discussion

This study investigated the association between MCH care and neonatal mortality in Angola. To the best of our knowledge, an association between inadequate MCH care and neonatal death has not been reported previously in Angola. This study showed that among mothers who received none or only one tetanus injection during the last pregnancy, none, one, two or three tetanus injections before the last pregnancy was associated with late neonatal mortality compared to five or more tetanus injections before the last pregnancy. No postnatal health check for children before discharge was associated with the occurrence of early and late neonatal death. Additionally, during the first 2 days after childbirth, no observation of breastfeeding, no counseling on breastfeeding, and no counseling on newborn danger signs were associated with early neonatal death. Furthermore, during the first 2 days after childbirth, no measurement of temperature and no examination of the cord were associated factors of early neonatal death.

The rate of early neonatal death was 22 per 1000 births, and that of neonatal death was 24 per 1000 births, consistent with the Angola DHS 2015–16 report [12].

In this study, receiving none, one, two or three tetanus vaccinations before the last pregnancy when received none or only one vaccination during the last pregnancy, was found to be associated with late neonatal death. This finding aligns with previous research [10]. However, among mothers who received none or only one tetanus injection, only 1.0% of them received five or more doses of tetanus vaccination prior to the last pregnancy, despite this being recommended by WHO guidelines [9]. Additionally, this result should be interpreted with caution, as excessive doses of the vaccine may lead to adverse effects. Receiving five or more doses of tetanus vaccine includes having received more than six doses of the vaccination, which exceeds the recommended doses [6]. Angola remains one of 12 countries that have not achieved the WHO strategy to eliminate maternal and neonatal tetanus [16, 17]. Increasing the coverage of tetanus vaccination is an important part of the strategy [18]. Since coverage of 4+ antenatal care is low, it is a background reason for insufficient tetanus vaccination uptake in Angola. Therefore, promotion of continuous care visits is required. Experience of women affects their continuous visits to ANC [19, 20]; therefore, quality of care needs to be ensured.

According to our study result, neonates who did not have their health checked before discharge were at greater risk of death. However, majority of neonatal deaths are caused by maternal complications, asphyxia and prematurity [21, 22]. In those cases, neonates are already in vulnerable condition at birth and not eligible for PNC, such as health check and counseling. Therefore, there is a possibility that care utilization is overestimated which may affected the correlation. However, considering that most mothers actually discharge from hospitals on the day or next day of delivery in Angola, receiving PNC before discharge possibly affects neonatal death in some cases. Also considering such low coverage of child health check before discharge (60%) [12], it is important to ensure availability and delivery of quality PNC.

Neonates born to mothers who had a chance to have breastfeeding observed and were counseled on breastfeeding during the first 2 days after childbirth were at less risk of early neonatal death. Early breast milk, especially colostrum, strengthens neonates’ immune system and protects them from infections, such as pneumonia and diarrhea [23–25]. Furthermore, exclusive breastfeeding prevents infants from drinking contaminated water, which often causes diarrhea [26]. A total of 2% and 9% of Angola’s neonates still died from diarrhea and pneumonia reported in 2020, respectively [27]. Nutritional education is an important part of postnatal care [5]; however, its implementation is sometimes challenging due to several reasons, such as nurse’s competency [28, 29] and attitude [30].

Neonates born to mothers who had counseling on newborn danger signs, and those who had their temperature measured and cord examined during the first 2 days after childbirth had less risk of early neonatal mortality. Disorders of body temperature [31, 32] and the cord [33] may result in adverse outcomes. Supporting mothers with information of newborns danger signs is crucial for them to acquire medical care in case of abnormalities.

The study results imply that improving service uptake and appropriate service delivery are crucial to addressing such high neonatal mortality. Low service utilization is probably caused by unavailability of quality care [8, 9]. In addition, barriers to MCH care utilization need to be identified. Unfortunately, we could not find the necessary variables, such as transportation to health facilities and human resources, to perform the analysis. Therefore, further studies are required to identify these barriers. We believe that our study assists nurses and midwives, who are primarily responsible for service delivery, as well as facility managers and policymakers, in recognizing that there is room for improving the availability of quality care. The situation of MCH services in Angola has likely evolved during the 9 years since the publication of the Angola DHS 2015–16. While the prolonged wars and conflicts in Angola ended in 2002, the healthcare system continues to undergo improvements. The COVID-19 pandemic may also have impacted the quality and availability of health services. With the upcoming release of a new Angola DHS, it is important to assess both the changes and the current state of health services, as well as related clinical outcomes.

### Limitations

There are some limitations to using the Angola DHS 2015–16. First, some important variables which influence neonatal conditions, such as asphyxia, pregnancy complications and premature birth, were not found in Angola DHS 2015–16. Therefore, influence of those variables to our multivariable logistic regression models is unknown. Second, the timing of tetanus injections before the last pregnancy was not known, which may have led to an overestimation of the number of mothers who received five or more injections among those who had none or only one tetanus injection during the last pregnancy. Third, further investigations or procedures following the collection of urine and blood samples, as well as blood pressure measurements, were unfortunately not investigated in the Angola DHS 2015–16. Therefore, related MCH care may be overestimated in this study. Fourth, due to the nature of cross-sectional survey, causality of relationship cannot be established. Fifth, there may have been recall bias on the date of the neonatal death and MCH care utilization. At last, the response “don’t know” was categorized as “never” or “no” for each relevant variable, which may have caused overestimation of “never” and “no”. Despite these limitations, the Angola DHS 2015–16 allowed us to attain the study objectives because it contained nationally representative clinical data and included mothers and neonates who did not utilize MCH care.

## Conclusions

Doses of maternal tetanus vaccination and child health check before discharge were modifiable factors associated to late neonatal death. In addition, no observation of breastfeeding, no breastfeeding counseling, no counseling of child danger signs, no measurement of child body temperature and no umbilical cord examination during 2 days after childbirth, as well as not checking child health before discharge, were associated with early neonatal death though the majority of early neonatal deaths occur within a few days after birth and these newborns may not have the opportunity to receive such care. Further studies to improve MCH care coverage are needed.

## Data Availability

Data are available upon request made to MEASURE DHS (URL: http://www.dhsprogram.com).

## Abbreviations

WHO: World Health Organization
MCH: Maternal and child health
ANC: Antenatal care
SP: Sulfadoxine and pyrimethamine
OR: Odds ratio
aOR: Adjusted odds ratio
CI: Confidential interval
